# Supplementary material for: Drivers with and without Obesity Respond Differently to a Multi-Component Health Intervention in Heavy Goods Vehicle Drivers
Source: Int J Environ Res Public Health. 2022 Nov 23;19(23):15546. doi: 10.3390/ijerph192315546 (PMC9739045; doi:10.3390/ijerph192315546)
Supplement: Supplementary file 1 [file ijerph-19-15546-s001.zip › ijerph-1995420-Supplementary.pdf]

Supplementary Table S1

Physical activity and sitting behaviours on workdays for participants with and without obesity based on BMI

Physical activity marker on workdays	Participants without obesity based on baseline BMI (BMI<30 kg/m²) N=90				Participants with obesity based on baseline BMI (BMI≥30 kg/m²) N=81			
	Change from baseline (Mean (SD))		Intervention effect* (95% CI)	p-value	Change from baseline (Mean (SD))		Intervention effect* (95% CI)	p-value
	Intervention n=36	Control n=54			Intervention N=38	Control N=43		
Steps/ day	-907 (2679)	-243 (2040)	-558.66 (-1513.99, 396.67)	0.252	186 (2393)	-465 (1972)	929.32 (78.31, 1780.33)	**0.032**
Time spent sitting (min/ day)	19.27 (109.11)	15.48 (92.75)	20.16 (-4.20, 44.53)	0.105	-33.74 (78.26)	31.39 (75.5)	-44.98 (-68.48, -21.49)	**<0.001**
Sitting bouts >30min (min/ day)	22.00 (137.10)	10.56 (129.69)	3.59 (-41.83, 49.01)	0.877	-38.98 (107.13)	32.66 (82.20)	-61.80 (-96.21, -27.38)	**<0.001**
Time spent standing (min/ day)	-13.66 (57.44)	4.36 (59.12)	-11.29 (-31.45, 8.88)	0.273	2.04 (61.12)	-19.62 (45.09)	24.53 (4.45, 44.61)	**0.017**
Time spent stepping (min/ day)	-11.02 (29.76)	-2.46 (24.72)	-6.84 (-17.98, 4.30)	0.229	3.99 (25.96)	-6.21 (23.19)	13.36 (3.57, 23.16)	**0.007**
Sit to upright transitions (n)	1.16 (28.37)	-0.02 (13.68)	2.51 (-5.88, 10.90)	0.557	-0.61 (11.78)	-1.53 (15.07)	2.82 (-2.74, 8.38)	0.320
Time spent in MVPA (min/ day)	-1.60 (12.80)	-0.82 (7.26)	-0.74 (-4.38, 2.90)	0.690	0.25 (15.15)	-0.86 (9.22)	2.57 (-1.60, 6.75)	0.227
Time spent in LPA (min/ day)	-9.42 (26.97)	-1.65 (23.73)	-6.17 (-16.31, 3.98)	0.234	3.75 (21.42)	-5.35 (20.42)	11.23 (2.25, 20.22)	**0.014**

*Change in intervention relative to control adjusted for variable at baseline and change in valid waking wear time from baseline to 6 months follow-up, and cluster size category (Small <40; Large ≥40)

Abbreviation: CI= Confidence Interval; LPA= Light physical activity; MVPA= Moderate to vigorous physical activity; SD= Standard deviation

Supplementary Table S2

Physical activity and sitting behaviours on non-workdays for participants with and without obesity based on BMI

Physical activity marker on non-workdays	Participants without obesity based on baseline BMI (BMI<30 kg/m²) N=90				Participants with obesity based on baseline BMI (BMI≥30 kg/m²) N=81			
	Change from baseline (Mean (SD))		Intervention effect* (95% CI)	p-value	Change from baseline (Mean (SD))		Intervention effect* (95% CI)	p-value
	Intervention N=36	Control N=54			Intervention N=38	Control N=43		
Steps/ day	-555 (3308)	-1039 (5682)	1045.85 (-417.13, 2508.84)	0.161	781 (4299)	-2229 (3525)	2467.13 (1040.89, 3893.40)	**0.001**
Time spent sitting (min/ day)	11.20 (111.05)	12.05 (100.98)	12.23 (-19.80, 44.25)	0.454	-17.42 (133.50)	59.69 (117.12)	-86.81 (125.36, -48.25)	**<0.001**
Sitting bouts >30min (min/ day)	11.95 (121.88)	25.52 (123.56)	-0.02 (-42.11, 42.07)	0.999	-8.73 (111.24)	73.51 (154.39)	-92.64 (-139.75, -45.52)	**<0.001**
Time spent standing (min/ day)	-11.05 (60.50)	-4.41 (68.21)	-7.71 (-33.47, 18.05)	0.558	20.89 (98.33)	-36.56 (58.37)	58.59 (25.85, 91.34)	**<0.001**
Time spent stepping (min/ day)	-6.73 (37.34)	-9.07 (54.00)	7.36 (-8.29, 23.02)	0.357	7.99 (43.78)	-27.05 (39.68)	29.81 (14.67, 44.95)	**<0.001**
Sit to upright transitions (n)	1.31 (15.79)	0.74 (17.10)	1.20 (-5.24, 7.63)	0.716	-1.04 (14.04)	-5.53 (14.71)	2.074 (-2.42, 6.57)	0.366
Time spent in MVPA (min/ day)	-1.77 (18.49)	-8.17 (49.18)	9.85 (2.27, 17.43)	**0.011**	7.34 (31.29)	-2.65 (12.54)	9.17 (0.39, 17.95)	**0.041**
Time spent in LPA (min/ day)	-4.96 (29.55)	-0.90 (23.65)	-1.04 (-11.28, 9.20)	0.842	0.66 (27.85)	-24.40 (33.39)	20.38 (9.52, 31.23)	**<0.001**

*Change in intervention relative to control adjusted for variable at baseline and change in valid waking wear time from baseline to 6 months follow-up, and cluster size category (Small <40; Large ≥40)

Abbreviations: CI= Confidence Interval; LPA= Light physical activity; MVPA=Moderate to vigorous physical activity; SD= Standard deviation

Supplementary Table S3

Physical activity and sitting behaviours measured across all valid days for participants with and without obesity based on Bodyfat %

	Participants without obesity based on baseline bodyfat percentage (bodyfat % < 25%) N=76				Participants with obesity based on baseline bodyfat percentage (bodyfat % ≥ 25%) N=131			
**Physical activity marker overall**	**Change from baseline (Mean (SD))**		**Intervention effect* (95% CI)**	**p-value**	**Change from baseline (Mean (SD))**		**Intervention effect* (95% CI)**	**p-value**
	**Intervention N=28**	**Control N=48**			**Intervention N=61**	**Control N=70**		
Steps/ day	-812 (2444)	-277 (1396)	-238.54 (-1260.47, 783.39)	0.647	511 (3007)	-1030 (2456)	1516.78 (746.08, 2287.48)	**<0.001**
Time spent sitting (min/ day)	2.90 (62.44)	11.41 (85.44)	1.22 (-21.96, 24.40)	0.918	-15.96 (82.26)	27.76 (73.83)	-40.50 (-60.03, -20.97)	**<0.001**
Sitting bouts >30min (min/ day)	24.47 (79.05)	21.20 (98.25)	-3.20 (-38.95, 32.56)	0.861	-16.65 (90.30)	28.42 (88.39)	-50.73 (-74.95, -26.51)	**<0.001**
Time spent standing (min/ day)	-1.48 (32.95)	-3.49 (36.79)	2.26 (-14.07, 18.58)	0.786	3.05 (53.51)	-14.57 (36.48)	21.38 (6.53, 36.22)	**0.005**
Time spent stepping (min/ day)	-10.58 (27.86)	-3.29 (17.57)	-3.56 (-15.67, 8.55)	0.564	5.31 (31.28)	-12.10 (25.43)	18.12 (9.83, 26.41)	**<0.001**
Sit to upright transitions (n)	-5.37 (11.77)	-1.73 (10.56)	-0.768 (-5.46, 3.92)	0.748	1.71 (13.73)	-1.44 (13.13)	3.68 (-0.57, 7.92)	0.090
Time spent in MVPA (min/ day)	-0.76 (13.47)	-0.12 (6.64)	1.44 (-2.68, 5.56)	0.494	4.14 (20.93)	-3.97 (17.50)	6.76 (1.74, 11.77)	**0.008**
Time spent in LPA (min/ day)	-9.83 (24.80)	-3.17 (17.74)	-2.97 (-13.33, 7.40)	0.575	1.17 (23.09)	-8.13 (19.34)	11.04 (4.48, 17.61)	**0.001**

*Change in intervention relative to control adjusted for variable at baseline and change in valid waking wear time from baseline to 6 months follow-up, and cluster size category (Small <40; Large ≥40)

Abbreviations: CI= Confidence Interval; LPA= Light physical activity; MVPA=Moderate to vigorous physical activity; SD= Standard deviation

Supplementary Table S4

Physical activity and sitting behaviours measured across all valid days for participants with and without obesity based on waist circumference

	Participants without obesity based on baseline waist circumference (waist circumference < 102cm) N=94				Participants with obesity based on baseline waist circumference (waist circumference ≥ 102cm) N=113			
**Physical activity marker overall**	**Change from baseline (Mean (SD))**		**Intervention effect* (95% CI)**	**p-value**	**Change from baseline (Mean (SD))**		**Intervention effect* (95% CI)**	**p-value**
	**Intervention N=42**	**Control N=52**			**Intervention N=47**	**Control N=66**		
Steps/ day	-692 (2716)	-442 (2412)	288.11 (-689.56, 1265.78)	0.564	798 (2895)	-946 (1838)	1585.71 (791.93, 2379.49)	**<0.001**
Time spent sitting (min/ day)	-0.19 (58.18)	7.67 (87.38)	1.34 (-19.84, 22.51)	0.902	-18.82 (89.88)	31.70 (70.24)	-44.61 (-66.14, -23.07)	**<0.001**
Sitting bouts >30min (min/ day)	15.81 (71.71)	8.85 (107.71)	2.86 (-27.46, 33.18)	0.853	-21.16 (98.82)	38.59 (76.15)	-61.47 (-87.70, -35.25)	**<0.001**
Time spent standing (min/ day)	-4.30 (30.26)	-0.89 (37.10)	-1.69 (-16.54, 13.17)	0.824	6.92 (59.19)	-17.29 (35.27)	25.34 (8.46, 42.23)	**0.003**
Time spent stepping (min/ day)	-9.65 (27.84)	-5.42 (24.57)	0.45 (-9.93, 10.83)	0.932	9.21 (31.22)	-10.96 (21.34)	19.40 (10.53, 28.28)	**<0.001**
Sit to upright transitions (n)	-4.10 (10.39)	-1.44 (10.57)	-1.36 (-5.34, 2.61)	0.502	2.68 (15.16)	-1.66 (13.27)	4.95 (-0.14, 10.04)	0.056
Time spent in MVPA (min/ day)	0.96 (20.35)	-1.73 (16.82)	5.41 (-0.86, 11.68)	0.091	4.06 (17.73)	-2.94 (11.86)	5.40 (0.67, 10.13)	**0.025**
Time spent in LPA (min/ day)	-10.62 (22.53)	-3.69 (18.42)	-3.33 (-11.86, 5.21)	0.445	5.15 (23.13)	-8.02 (18.99)	13.45 (6.20, 20.70)	**<0.001**

*Change in intervention relative to control adjusted for variable at baseline and change in valid waking wear time from baseline to 6 months follow-up, and cluster size category (Small <40; Large ≥40)

Abbreviations: CI= Confidence Interval; LPA= Light physical activity; MVPA=Moderate to vigorous physical activity; SD= Standard deviation

Supplementary Table S5

Cardiometabolic and lifestyle secondary outcome changes from baseline to 6 months follow up in participants with and without obesity (based on bodyfat %)

	**Participants without obesity based on baseline bodyfat percentage (bodyfat % < 25%)** N=93				**Participants with obesity based on baseline bodyfat percentage (bodyfat % ≥ 25%)** N=151			
**Anthropometric measures**	**Change from baseline (Mean (SD))**		**Intervention effect* (95% CI)**	**p-value**	**Change from baseline (Mean (SD))**		**Intervention effect* (95% CI)**	**p-value**
	**Intervention N=34**	**Control N=58**			**Intervention N=67**	**Control N=84**		
Body fat (%)	0.46 (1.65)	0.29 (2.11)	0.29 (-0.43, 1.00)	0.428	-0.64 (2.41)	-0.19 (1.92)	-0.53 (-1.22, 0.17)	0.141
Weight (kg)	-0.81 (3.63)	0.08 (4.22)	-0.74 (-2.28, 0.80)	0.346	-1.87 (5.83)	-0.34 (4.91)	-1.81 (-3.47, -0.16)	**0.032**
BMI (kg/m^2)	-0.06 (1.05)	0.04 (1.19)	-0.06 (-0.50, 0.38)	0.794	-0.54 (1.79)	-0.07 (1.53)	-0.56 (-1.08, -0.03)	**0.037**
Waist Circumference (cm)	-1.11 (5.41)	-0.06 (5.26)	-0.84 (-3.03, 1.34)	0.450	-1.45 (7.09)	-0.22 (5.37)	-1.77 (-3.74, 0.19)	0.077
Hip Circumference (cm)	-0.83 (3.31)	-0.17 (4.13)	0.06 (-1.41, 1.53)	0.938	-1.00 (4.03)	0.05 (4.74)	-1.29 (-2.61, 0.03)	0.056
Neck Circumference (cm)	-0.14 (1.54)	0.48 (1.29)	-0.56 (-1.17, 0.05)	0.073	-0.29 (2.02)	0.42 (2.06)	-0.71 (-1.33, -0.09)	**0.024**
Grip strength (kg)	0.20 (7.28)	0.46 (5.77)	-0.44 (-3.16, 2.29)	0.753	1.04 (4.25)	-0.16 (5.61)	1.29 (-0.30, 2.87)	0.112
**Blood pressure**								
Systolic Blood pressure (mm Hg)	-3.16 (10.78)	-2.05 (11.09)	0.53 (-3.93, 4.98)	0.817	-2.26 (10.52)	-2.28 (13.90)	-1.10 (-4.86, 2.67)	0.567

Diastolic Blood pressure (mm Hg)	-1.44 (9.42)	-0.30 (8.48)	0.16 (-3.14, 3.45)	0.927	-0.93 (7.33)	-1.05 (8.73)	-0.50 (-2.93, 1.93)	0.687
Heart rate (beats/ min)	-1.90 (10.97)	1.35 (8.32)	-3.71 (-7.08, -0.33)	**0.031**	-2.05 (9.52)	-1.55 (9.45)	-0.19 (-2.86, 2.49)	0.892
**Blood markers**								
HbA1c (mmol/ mol)	-0.63 (4.36)	0.87 (5.09)	-1.63 (-3.37, 0.10)	0.065	-0.30 (7.72)	0.17 (6.66)	-0.94 (-3.17, 1.28)	0.405
Triglycerides (mmol/L)	-0.01 (0.72)	0.05 (1.14)	-0.10 (-0.42, 0.21)	0.518	0.08 (0.97)	0.05 (0.87)	-0.01 (-0.29, 0.26)	0.919
HDL-Cholesterol (mmol/L)	0.03 (0.28)	0.00 (0.25)	0.05 (-0.05, 0.14)	0.313	0.10 (0.23)	0.04 (0.25)	0.07 (0.01, 0.13)	**0.016**
LDL-Cholesterol (mmol/L)	-0.19 (0.76)	-0.10 (0.88)	-0.15 (-0.45, 0.14)	0.309	0.10 (0.80)	0.06 (0.84)	-0.02 (-0.27, 0.22)	0.848
Total Cholesterol (mmol/L)	-0.14 (0.85)	-0.03 (0.93)	-0.14 (-0.47, 0.18)	0.401	0.19 (0.89)	0.11 (0.90)	0.03 (-0.24, 0.30)	0.826
**Lifestyle behaviours**								
Fruit intake grams/ day	4.99 (97.34)	41.72 (129.02)	-28.43 (-75.42, 18.55)	0.236	8.44 (165.96)	13.32 (129.73)	13.25 (-29.55, 56.04)	0.544
Vegetable intake grams/ day	-13.58 (145.95)	13.71 (142.20)	-7.41 (-53.25, 38.44)	0.752	34.50 (240.66)	-12.71 (185.70)	71.36 (10.69, 132.03)	**0.021**
Dietary Quality Score	0.38 (2.41)	0.21 (2.23)	-0.09 (-0.92, 0.74)	0.832	0.01 (2.56)	0.29 (2.21)	-0.387 (-0.99, 0.21)	0.205

*Change in intervention relative to control adjusted for variable at baseline and cluster size category (Small <40; Large ≥40)

Abbreviations: BMI= Body Mass Index; CI= Confidence Interval; HbA1c= Haemoglobin A1c; HDL= High density lipoprotein; LDL=Low density lipoprotein; SD= Standard deviation

Supplementary Table S6

Cardiometabolic and lifestyle secondary outcome changes from baseline to 6 months follow up in participants with and without obesity (based on waist circumference)

	**Participants without obesity based on baseline waist circumference (waist circumference < 102cm) N=112**				**Participants with obesity based on baseline waist circumference (waist circumference $\geq$ 102cm) N=132**			
**Anthropometric measures**	**Change from baseline (Mean (SD))**		**Intervention effect* (95% CI)**	**p-value**	**Change from baseline (Mean (SD))**		**Intervention effect* (95% CI)**	**p-value**
	**Intervention N=49**	**Control N=63**			**Intervention N=53**	**Control N=79**		
Body fat (%)	0.02 (1.98)	0.14 (2.34)	0.13 (-0.60, 0.86)	0.720	-0.52 (2.44)	-0.12 (1.68)	-0.47 (-1.22, 0.28)	0.223
Weight (kg)	-0.69 (3.29)	0.26 (3.58)	-0.692 (-1.84, 0.45)	0.235	-2.26 (6.40)	-0.51 (5.32)	-1.95 (-3.97, 0.07)	0.060
BMI (kg/m^2)	-0.08 (0.96)	0.08 (1.05)	-0.10 (-0.44, 0.24)	0.556	-0.64 (1.97)	-0.11 (1.62)	-0.59 (-1.22, 0.05)	0.072
Waist Circumference (cm)	0.05 (5.31)	0.46 (3.95)	-0.10 (-1.91, 1.70)	0.910	-2.62 (7.30)	-0.65 (6.16)	-2.34 (-4.63, -0.05)	**0.045**
Hip Circumference (cm)	-0.57 (3.22)	0.20 (3.31)	-0.07 (-1.23, 1.09)	0.903	-1.30 (4.23)	-0.23 (5.26)	-1.17 (-2.72, 0.39)	0.141
Neck Circumference (cm)	-0.18 (1.55)	0.55 (1.81)	-0.56 (-1.20, 0.07)	0.082	-0.29 (2.12)	0.36 (1.76)	-0.62 (-1.29, 0.04)	0.066
Grip strength (kg)	-0.01 (6.11)	0.99 (5.64)	-0.98 (-3.11, 1.15)	0.367	1.45 (4.74)	-0.62 (5.61)	2.13 (0.41, 3.85)	**0.015**
**Blood pressure**								
Systolic Blood pressure (mm Hg)	-3.11 (11.11)	-2.47 (11.28)	0.40 (-3.41, 4.21)	0.837	-2.07 (10.12)	-1.97 (13.94)	-0.82 (-4.76, 3.12)	0.683

Diastolic Blood pressure (mm Hg)	-0.78 (9.04)	-0.28 (8.39)	-0.04 (-2.80, 2.71)	0.976	-1.41 (7.13)	-1.11 (8.81)	-0.49 (-3.10, 2.12)	0.712
Heart rate (beats/ min)	-1.57 (10.60)	1.06 (9.15)	-2.57 (-5.65, 0.50)	0.101	-2.40 (9.47)	-1.50 8.93)	-0.55 (-3.39, 2.29)	0.704
**Blood markers**								
HbA1c (mmol/ mol)	0.10 (4.86)	1.16 (5.38)	-1.66 (-3.25, -0.07)	**0.041**	-0.89 (8.11)	-0.11 (6.53)	-0.97 (-3.40, 1.46)	0.435
Triglycerides (mmol/L)	0.06 (0.96)	0.12 (0.94)	-0.07 (-0.39, 0.24)	0.648	0.04 (0.83)	-0.01 (1.03)	-0.00 (-0.29, 0.29)	0.982
HDL-Cholesterol (mmol/L)	0.05 (0.26)	0.02 (0.25)	0.06 (-0.02, 0.13)	0.154	0.11 (0.24)	0.02 (0.26)	0.07 (0.00, 0.13)	**0.040**
LDL-Cholesterol (mmol/L)	-0.18 (0.78)	0.02 (0.85)	-0.24 (-0.51, 0.03)	0.079	0.16 (0.77)	-0.02 (0.87)	0.10 (-0.16, 0.36)	0.449
Total Cholesterol (mmol/L)	-0.11 (0.82)	0.10 (0.89)	-0.21 (-0.49, 0.07)	0.148	0.25 (0.93)	0.01 (0.93)	0.14 (-0.16, 0.43)	0.357
**Lifestyle behaviours**								
Fruit intake grams/ day	-12.68 (107.88)	34.91 (125.28)	-36.53 (-76.66, 3.61)	0.074	25.36 (172.50)	16.96 (133.45)	25.15 (-24.47, 74.76)	0.321
Vegetable intake grams/ day	-13.47 (197.38)	18.53 (180.12)	4.11 (-44.92, 53.14)	0.869	47.09 (225.92)	-18.23 (159.32)	76.81 (11.07, 142.55)	**0.022**
Dietary Quality Score	0.29 (2.63)	0.11 (2.19)	-0.20 (-0.91, 0.52)	0.591	0.00 (2.39)	0.37 (2.24)	-0.37 (-1.04, 0.30)	0.282

*Change in intervention relative to control adjusted for variable at baseline and cluster size category (Small <40; Large ≥40)

Abbreviations: BMI= Body Mass Index; CI= Confidence Interval; HbA1c= Haemoglobin A1c; HDL= High density lipoprotein; LDL= Low density lipoprotein; SD= Standard deviation

Supplementary Table S7

Physical activity and sitting behaviour changes from baseline to 16-18 months follow-up for participants with and without obesity based on BMI

	**Participants without obesity based on baseline BMI (BMI<30 kg/m²) N=94**				**Participants with obesity based on baseline BMI (BMI≥30 kg/m²) N=69**			
**Physical activity marker overall**	**Change from baseline (Mean (SD))**		**Intervention effect* (95% CI)**	**p-value**	**Change from baseline (Mean (SD))**		**Intervention effect* (95% CI)**	**p-value**
	**Intervention N=39**	**Control N=55**			**Intervention N=35**	**Control N=34**		
Steps/ day	-482.96 (2551.22)	-90.04 (2518.26)	-149.77 (-1089.20, 789.67)	0.755	-43.60 (3072.20)	-338.94 (1507.66)	239.21 (-826.69, 1305.11)	0.660
Time spent sitting (min/ day)	13.48 (78.98)	4.94 (86.09)	9.01 (-14.66, 32.68)	0.456	-12.78 (106.18)	1.78 (78.04)	-27.42 (-66.92, 12.07)	0.174
Time spent in MVPA (min/ day)	-0.29 (14.97)	-1.82 (16.91)	2.51 (-2.16, 7.17)	0.292	0.17 (19.44)	0.87 (10.67)	0.24 (-6.61, 7.08)	0.946
Time spent in LPA (min/ day)	-6.79 (25.41)	0.87 (19.58)	-5.26 (-14.47, 3.96)	0.264	0.04 (26.94)	-5.25 (14.75)	5.18 (-4.96, 15.33)	0.317

*Change in intervention relative to control adjusted for variable at baseline and change in valid waking wear time from baseline to 16-18 months follow-up, and cluster size category (Small <40; Large ≥40)

Abbreviations: CI= Confidence Interval; LPA= Light physical activity; MVPA=Moderate to vigorous physical activity; SD= Standard deviation

Supplementary Table S8

Lifestyle secondary outcome changes from baseline to 16-18 months follow-up in participants with and without obesity (based on BMI)

	Participants without obesity based on baseline BMI (BMI<30 kg/m²) N=115				Participants with obesity based on baseline BMI (BMI≥30 kg/m²) N=97			
**Lifestyle Behaviours**	**Change from baseline (Mean (SD))**		**Intervention effect* (95% CI)**	**p-value**	**Change from baseline (Mean (SD))**		**Intervention effect* (95% CI)**	**p-value**
	**Intervention N=51**	**Control N=64**			**Intervention N=50**	**Control N=47**		
Vegetable intake grams/ day	-58.61 (166.83)	1.37 (134.37)	-23.99 (-60.76, 12.78)	0.201	-17.03 (146.93)	10.70 (168.57)	-13.45 (-64.99, 38.10)	0.609
Fruit intake grams/ day	2.66 (125.77)	-10.76 (110.41)	29.14 (-12.04, 70.32)	0.165	-33.72 (194.37)	8.43 (99.11)	-12.56 (-63.92, 38.79)	0.632
Dietary Quality Score	0.67 (2.27)	0.27 (2.17)	0.38 (-0.25, 1.01)	0.242	-0.18 (2.29)	0.00 (1.92)	-0.39 (-0.98, 0.20)	0.193

*Change in intervention relative to control adjusted for variable at baseline and cluster size category (Small <40; Large ≥40)

Abbreviations: BMI= Body Mass Index; CI= Confidence Interval; SD= Standard deviation
